# Who Is a Pathologist According to Oncology Patients and Internet Users? A Survey Study

**DOI:** 10.1007/s13187-019-01640-0

**Published:** 2019-10-30

**Authors:** Michał Kunc, Kevin Miszewski, Michał Bieńkowski, Alexandra Kamieniecki, Marcin Ekman, Adam Gorczyński, Wojciech Biernat

**Affiliations:** 1 Department of Pathology, Medical University of Gdańsk, Mariana Smoluchowskiego 17, 80-214 Gdańsk, Poland; 2 Student Scientific Circle of Pathomorphology, Medical University of Gdańsk, Gdańsk, Poland; 3 Department of Surgical Oncology, Medical University of Gdańsk, Gdańsk, Poland

**Keywords:** Pathologists, Survey, Patients, Internet

## Abstract

The pathologist is frequently called “the doctor’s doctor.” However, there are many uncertainties about the role of a pathologist among patients and policymakers and even among other medical specialties. The aim of the current study is to analyze the misconceptions of who a pathologist is among inpatients and Internet users, to find where the lack of understanding is originating from, and to confirm the need to educate the general public about pathologists. The survey of Internet users was conducted among Facebook users, utilizing the snowball sampling method. Inpatients were randomly recruited in the Department of Surgical Oncology. Seventy-eight inpatients and 320 Internet users were enrolled in the study. Significantly, more hospital patients than Internet users answered that the pathologist is not an MD (*p* = 0.00953). A portion of participants stated that pathologists do not make diagnoses (*n* = 28, 7.03%) and do not influence the treatment plan (*n* = 37, 9.30%) and that the other specialists do not gain anything from the pathologist’s work (*n* = 67, 16.83%). Only 15.07% of respondents had their information about pathologists from other doctors. The findings from this study should show that even the most basic knowledge of a pathologist being an MD is not known. Pathologists are not recognized for being involved in the diagnosis of diseases. This should provide an incentive to pathologists to teach future doctors, policymakers, and patients about the perplexity of the pathology specialty. It shows obvious gaps in the knowledge of the treatment process as a whole.

## Introduction

The pathologist is frequently called “the doctor’s doctor” [1]. Pathologists are involved in establishing the definitive diagnosis and the treatment planning of patients with some of the most complicated diseases. They typically have the most variable collection of cases in the back of their minds as their differential diagnosis and have extensive knowledge of almost all diseases. Pathologists teach students, doctors, and patients about the basis of diseases. They cooperate with primary care physicians in order to provide the best treatment plans for patients.

There are many uncertainties about the role of a pathologist among patients and policymakers and even among other medical specialties [2]. Some pathologists spend most of their time behind microscopes or working in laboratories and may have little to no patient contact. Some pathologists prefer being in the background, but others feel as though patient interaction is missing. People not associated with the medical field may not know of the pathologist’s existence, unless they need a microscopic diagnosis or have family members that have had cancer. Some patients that know of the pathologist may think that he would be of no help to them, because of the misconception that pathologists only deal with autopsies (forensic and clinical).

Pathologists have grown accustomed to not interacting with patients and are more comfortable communicating with other pathologists [2]. Even though it is still rare for them to communicate directly with patients, social media may be the bridge needed to start better pathologist-to-patient communication answering the questions and doubts about basic disease processes. This could lead to implicating real-life communication in hospitals in the future. On the other hand, do patients know anything about pathologists? If the pathologist is not recognized as an MD, what does the general public think a pathologist does? Are the common stereotypes about pathologists the same around the world? We have conducted a survey of basic questions about who a pathologist is, the role of a pathologist in medicine and the treatment of disease. The aim of the current study is to analyze the misconceptions of who a pathologist is among inpatients and Internet users, to find where the lack of understanding is originating from, and to confirm the need to educate the general public about pathologists.

## Material and Methods

The survey aiming to investigate the knowledge about pathologists in inpatients and Internet users was designed through a discussion among the co-authors. The final version was reviewed and accepted by medical students (*n* = 2), surgery resident (*n* = 1), pathology residents (*n* = 3), and an experienced pathology specialist (*n* = 1). The final version of the questionnaire consisted of 9 single-choice and 2 multiple-choice questions divided into groups aiming to gather (1) demographic data (sex, age, education, occupational association with medicine) (Table 1); (2) the knowledge of the role of pathologists in medicine; and (3) the sources of information about the pathologists. The questions are included in Tables 2, 3, and 4. The questionnaire form was anonymous.

**Table 1:** The basic demographic data of the study groups

		Patients, *n* (%)	Internet users, *n* (%)
Sex	Female	46 (58.97)	257 (80.31)
	Male	32 (41.03)	63 (19.69)
Age (years)	18–39	8 (10.26)	284 (88.75)
	40–59	14 (17.95)	31 (9.69)
	60–74	33 (42.31)	4 (1.25)
	> 74	23 (29.49)	1 (0.31)
Education	Low	11 (14.10)	5 (1.57)
	Medium	40 (51.28)	133 (41.69)
	High	27 (34.62)	181 (56.74)

**Table 2:** Questions included in the survey study

Question	Patients, *n* (%)	Internet, *n* (%)	p*
Who is a pathologist?			
Medical doctor	39 (50.00)	234 (73.82)	0.00953
Medical technician	9 (11.54)	28 (8.83)	
Biologist	3 (3.85)	4 (1.26)	
Biotechnologist	7 (8.97)	22 (6.94)	
Bio-medical analyst	20 (25.64)	29 (9.15)	
A pathologist mainly deals with autopsies.			
True	27 (33.33)	188 (58.75)	0.00396
False	54 (66.67)	132 (41.25)	
Pathologists often cooperate with the police in cases where a murder is suspected.			
Strongly agree	23 (28.40)	35 (11.04)	0.03864
Agree	18 (22.22)	108 (34.07)	
I don’t know	24 (29.63)	95 (29.97)	
Disagree	11 (13.58)	51 (16.09)	
Strongly disagree	5 (6.17)	28 (8.83)	
Most physicians use the work that a pathologist does.			
Strongly agree	22 (28.21)	56 (17.67)	0.4699
Agree	25 (32.05)	124 (39.12)	
I don’t know	26 (33.33)	75 (23.66)	
Disagree	4 (5.13)	57 (17.98)	
Strongly disagree	1 (1.28)	5 (1.58)	
The work done by a pathologist has significant influence on the choice of treatment for patients.			
Strongly agree	24 (30.77)	142 (44.79)	0.3888
Agree	26 (33.33)	120 (37.85)	
I don’t know	16 (20.51)	30 (9.46)	
Disagree	8 (10.26)	15 (4.73)	
Strongly disagree	4 (5.13)	10 (3.15)	
The pathologist plays a big role in making the correct diagnosis of a disease.			
Strongly agree	28 (35.90)	172 (54.26)	3.549411E−06
Agree	21 (26.92)	105 (33.12)	
I don’t know	21 (26.92)	20 (6.31)	
Disagree	4 (5.13)	18 (5.68)	
Strongly disagree	4 (5.13)	2 (0.63)

**Table 3:** Answers of the participants when ask on what are the duties of a pathologist

What are the duties of a pathologist?		Patients, *n* (%)	Internet, *n* (%)	p*
Microscopic assessment of tissues	Yes	52 (66.67)	288 (90.00)	< 0.001
	No	26 (33.33)	32 (10.00)	
Describing radiological images	Yes	9 (11.54)	27 (8.44)	1.0
	No	69 (88.46)	293 (91.56)	
Autopsies	Yes	25 (32.05)	188 (58.75)	0.0002
	No	53 (67.95)	132 (41.25)	
Cooperation with the police while inspecting crime scenes	Yes	10 (12.82)	78 (24.37)	0.2471
	No	68 (87.18)	242 (75.63)	
Biopsies	Yes	18 (23.08)	114 (35.63)	0.3132
	No	60 (76.92)	206 (64.37)	
Performing blood tests	Yes	6 (7.69)	40 (12.5)	1.0
	No	72 (92.31)	280 (87.5)	
Operations	Yes	8 (10.26)	5 (1.56)	0.001
	No	70 (89.74)	315 (98.44)	
Treating chronic diseases	Yes	5 (6.41)	8 (2.50)	0.7355
	No	73 (93.59)	312 (97.50)	
I do not know	Yes	12 (15.38)	21 (6.56)	0.1016
	No	66 (84.62)	299 (93.44)

**Table 4:** Answers of the participants when ask on their primary sources of information about pathologists

What is your primary source of information about pathologists?		Patients, *n* (%)	Internet, *n* (%)	p*
Doctors	Yes	12 (15.38)	48 (15.00)	1.0
	No	66 (84.61)	272 (85.00)	
Family	Yes	57 (73.07)	44 (13.75)	0.0382
	No	21 (26.92)	276 (86.25)	
Friends	Yes	70 (89.74)	253 (79.06)	0.2444
	No	8 (10.26)	67 (20.94)	
Internet	Yes	25 (32.05)	195 (60.94)	< 0.001
	No	53 (67.95)	125 (39.06)	
Books	Yes	19 (24.36)	110 (34.38)	0.7211
	No	59 (75.64)	210 (65.62)	
Television	Yes	24 (30.77)	43 (13.44)	0.002
	No	54 (69.23)	277 (86.56)	
Radio	Yes	4 (5.13)	3 (0.94)	0.0926
	No	74 (94.87)	317 (99.06)	
None of the above	Yes	1 (1.67)	42 (13.12)	0.1082
	No	59 (98.33)	278 (86.88)	

The survey of Internet users was conducted among Facebook users with the use of the platform Google Forms, utilizing the snowball sampling method between June and August 2018. Inpatients were randomly recruited in the Department of Surgical Oncology. Only patients in good general condition who freely agreed to fill the questionnaire during hospitalization were enrolled. Only adults (> 18 years old) were included in the study. All respondents with the occupation in medical field were excluded from the study.

### Statistics

The statistical analysis was performed using R version 3.5.1 [3]. All single-choice questions were analyzed using frequency tables and chi-squared test. Additionally, ordinal-level questions were compared between groups using Mann-Whitney-Wilcoxon test or Kruskal-Wallis test (depending on the number of groups) with Bonferroni correction for multiple testing. Questions with more than two options were investigated with correspondence analysis using the “ca” package [4]. Multiple-choice questions were analyzed with Test for Multiple Marginal Independence with 1000 bootstrap iterations, second-order Rao-Scott adjustment and Bonferroni correction for the multiple testing using the “MRCV” package [5].

### Ethics

Institutional guidelines regarding survey studies were followed.

## Results

A total of 650 questionnaires were collected, including 81 inpatients and 569 Internet users. After exclusion of respondents who were occupied in medical field, 78 inpatients and 320 Internet users were enrolled in the study. The basic demographic data of the study groups is shown in Table 1.

### Essential Knowledge About the Role of Pathologist in Medicine

Significantly more inpatients than Internet users answered that the pathologist is not an MD. The number of patients who chose MD and non-doctor options (biotechnologist, biologist, medical technician, bio-medical analyst) was the same (*n* = 39, 50.00%). In the latter group, bio-medical analyst was the most frequent answer (20, 25.64%). On the other hand, the majority of the Internet users answered correctly to this question (*n* = 234, 73.82%), and it correlated with higher education and younger age.

A portion of participants stated that pathologists do not make diagnoses (*n* = 28, 7.03%) and do not influence the treatment plan (*n* = 37, 9.30%) and that the other specialists do not gain anything from the pathologist’s work (*n* = 67, 16.83%). Moreover, patients frequently admitted that they did not know the answers to these questions. Internet users were more skeptic to the statement that most doctors utilize the effects of pathologists’ work; however, it was not statistically significant. Interestingly, most Internet users (58.75%) answered that autopsy is the main part of pathologists’ job compared with only 33.33% of patients. On the other hand, patients more frequently thought that pathologists cooperate with police or prosecutors during investigations of homicides. Despite the fact that only 68.59% (*n* = 273) of the respondents were able to distinguish pathology as a medical specialty, we found that 85.43% (*n* = 340) of respondents were still able to associate this specialty with a microscope.

### The Pathologists’ Duties and the Sources of Information About the Pathologists

**Fig. 1:**
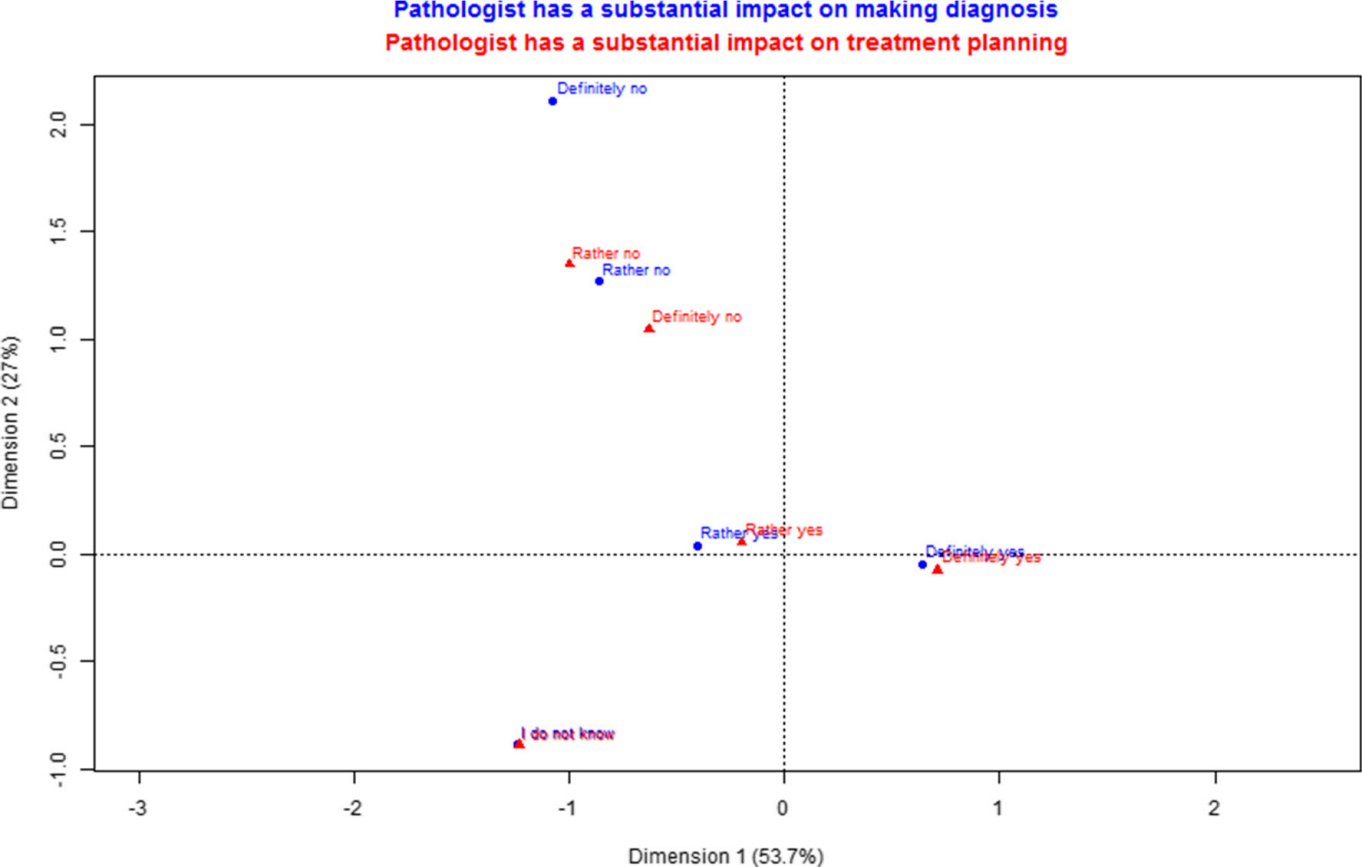
Correspondence analysis between “Pathologist has a substantial impact on making diagnosis” (blue) and “Pathologist has a substantial impact on treatment planning” (red). The distance between red and blue points gives a measure of the relationships between answers to two different questions. Summary of dimension 1 and dimension 2 represents the percentage of the variance in the data which is displayed on the chart

Generally, Internet users had better knowledge than inpatients about the pathologists’ duties (Table 3). Intriguingly, 8 inpatients (10.26%) answered that pathologists perform surgeries, as compared with 5 Internet users (1.56%, *p* = 0.001). Inpatients were less capable to acknowledge the pathologist’s involvement in the diagnosis of the disease (62.03%) compared with Internet users (87.38%). Furthermore, respondents exhibit a constant tendency to sustain their specific view in the following questions regarding the duties of pathologists as seen in Fig. 1. Patients also less frequently recognize pathologists as working with microscopes and performing biopsies. However, on the contrary to expectations, tissue biopsy was recognized fairly often (33.17%) as a duty in general. In the whole study cohort, younger and higher educated participants had more correct answers than older participants. Respondents who never heard about pathologists tend to choose complete blood count testing as a duty (*p* = 0.0011). These participants tended to choose this option before autopsies. Having a history of cancer or family members working in a medical field was not associated with a better understanding of the role of a pathologist.

**Fig. 2:**
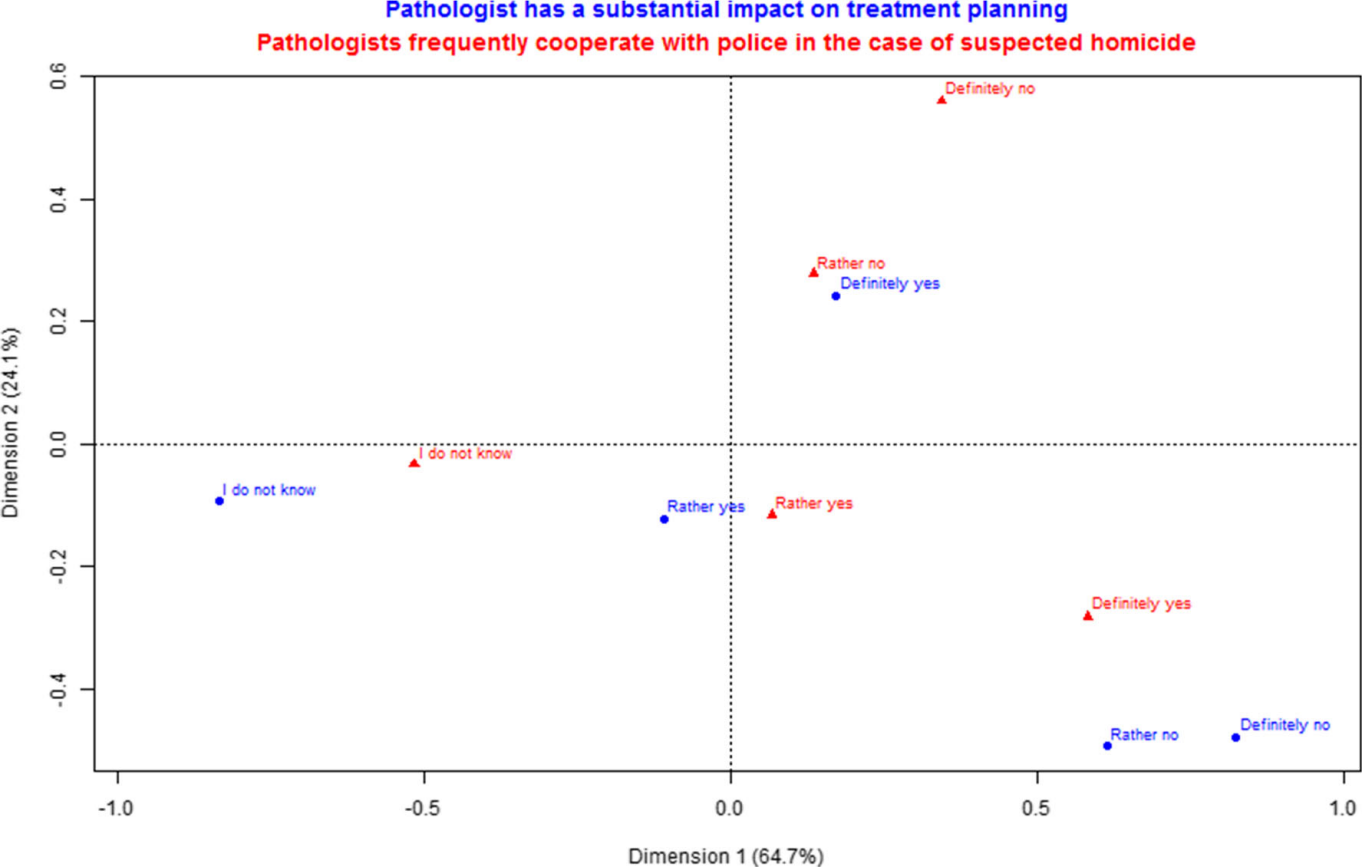
Correspondence analysis between “Pathologist has a substantial impact on treatment planning” (blue) and “Pathologists frequently cooperate with police in the case of suspected homicide” (red). The distance between red and blue points gives a measure of the relationships between answers to two different questions. Summary of dimension 1 and dimension 2 represents the percentage of the variance in the data which is displayed on the chart

The respondents who chose that pathologists frequently cooperate with police in the cases of homicide tended to answer that they also perform autopsies (*p* < 0.001). They also tended to not acknowledge the pathologist’s involvement in treatment planning (Fig. 2). Interestingly, there was an association between identification of a pathologist as a person involved in a murder investigation and books or television as a source of knowledge (*p* < 0.001). There were no substantial differences between sexes in terms of identifying the duties of a pathologist (*p* = 0.0895) and sources of information (*p* = 0.2176).

Only 15.07% of respondents said they gained their information about pathologists from other doctors. This source of information was the most effective in terms of correct answers. Family and friends were the main sources of knowledge for inpatients. Radio was the least common source of information. Older persons more frequently got their information about pathologists from the television, than the respondents aged 18–39 years. As anticipated, people with family members occupied in medical field more frequently chose family as a source of information (*p* = 0.0101).

## Discussion

The findings from our survey study show that the awareness of who a pathologist is still hidden in the background, only partially understood by Internet users and especially misconceived among inpatients. General findings which were identified in the current study include the following: (1) Many inpatients did not know that a pathologist is an MD with “bio-medical analyst” being the most common non-MD option chosen. In 15% of respondents, the information about pathologists was attained from other doctors, but when the doctor was the source of information, the answers were more correct. (2) Although only 68% of participants acknowledged pathologists as an MD, pathologists are still recognized for using microscopes in both inpatients and Internet users. (3) A history of cancer or family members working in the medical field were not associated with differences in understanding who a pathologist is.

It is thought that pathologists are only involved in autopsies and criminal investigations. People who chose these duties tended to get their information from books and TV sources. Internet users thought pathologists mainly deal with autopsies. The pathologist’s portrayal in the entertainment industry has been minimal and when present, vague and inaccurate with a propensity to accentuate forensics. One study showed that 27% of the patients involved in rare sarcoma support groups thought an oncologist makes the diagnosis of cancer [6], which is consistent with our study. There is still a lot of work to do in educating patients about the role of pathologists in patient care. Another study showed evidence that medical students also believe in this stereotype and that students believed 40% or more of typical pathologists’ caseloads consist of autopsy specimens (3rd year medical school longitudinal pathology curriculum) [7]. One way the stereotypes of pathology can be counterbalanced is by making sure medical students are aware of the pathologist’s role in medicine from the very beginning of their studies and putting emphasis on the fact that pathology benefits all specialties no matter who they want to become in the future. Wanting to become a surgeon in the future is a common dream among medical students, but very few want to become pathologists. One study revealed that the practice of pathology and its concrete application in patient care are not commonly addressed in American medical school curricula [8]. Pathology and medicine are mutually dependent and beneficial to one another, and could neither exist on its own. If future doctors are unaware of the pathologist’s role in medicine, the intricate picture of pathology will continue to be a mystery to most. This problem has been addressed by a group of pathologist creating program on Twitter called #Path2Path aiming to encourage medical students to choose pathology as a specialty.

Only 15% of patients received information about pathologists from doctors, and we concluded this group was more knowledgeable of the pathologist’s role. Most complaints about doctors are related to issues of communication, not clinical competency. Doctors with better communication and interpersonal skills are able to detect problems easier, can prevent medical crises and expensive intervention, and provide better support of their patients [1]. In our department, due to the relative shortage of clinicians in Poland, pathologists are frequently supposed to inform the patients about the diagnosis obtained from the histopathology or cytopathology report and especially in cases of malignancy. This is not a default scenario, but rather a consequence of the relative shortage of physicians in Poland and long queues to oncologists. Nevertheless, in such situation, the waiting time for diagnosis is reduced; patients receive detailed information about the diagnostic process, resection margins, and prognostic or predictive factors of their disease. Finally, the pathologist is acknowledged for being involved in patient management. Although this is true, more inpatients than Internet users did not know that a pathologist is an MD and admitted they did not know many answers to the questions.

A very recent study reported the first direct pathologist-patient consultation [9]. Pathologists conducted patient consultations reviewing biopsy or surgery findings on a multiheaded microscope or computer screen. Patients were curious to see their biopsies under the microscope or on the computer screen, instead of Googled images. Patients stated the desire to see normal tissue and compare it with their tumor. Later, patients completed a patient satisfaction survey. Early data suggests that the program may provide effective patient-specific education and may be a reliable source for some patients, although they stated some pathologists fear exposure to medicolegal lawsuits or felt that the commitment is too high and prefer to not change their practice [9]. Some pathologists at our center agreed that they would like to implement patient consultations, but others bluntly stated they chose the pathology specialty to avoid patients. Although this may be one of the problems encountered among the pathology specialty, it leads us to asking a few questions for future studies. Would patients in the future ask to speak with their pathologists if they knew of their existence? Would patients want a consult with their pathologist if they knew of the influence they have in the treatment planning of their disease?

Although only 68% of respondents distinguished pathology as a medical specialty, microscopes were in fact recognized as a tool used by pathologists by the majority of respondents. We think the reason for microscopes being recognized is that respondents tended to think pathologists are bio-medical analysts. One possible reason is that the general public only knows of these basic methods. The general public does not know of all the steps it takes in making a definitive diagnosis or the difficulties encountered along the way. It is not known that pathology is a very complex specialty that has multiple responsibilities and uses various tools for diagnosing diseases. Surprisingly in our study, 1/3 of participants recognized that pathologists perform biopsies. Some medical students from our university stated they did not realize pathologists perform biopsies until their later years in medical school or until they became directly involved with the pathology department after completing the required pathology course.

### Limitations of the Study

Due to the use of the snowball sampling method in Internet users, it is biased toward females. A relatively low number of inpatients were enrolled, when compared with Internet users. Moreover, there are some countries of origin-related issues in our study, which should be emphasized. In Poland, surgical pathology and forensic medicine are completely separate careers, and neither of them is related to blood transfusion/banking like in the USA. The term bio-medical analyst is used in Poland to describe a person with post-graduate training in the field of laboratory diagnostics, but is not an MD. Thus, some caution is indicated in drawing conclusions from this study.

## Conclusions

The findings from this study should show that even the most basic knowledge of a pathologist being an MD is not known. Pathologists are not recognized for being involved in diagnosis of diseases. This should provide an incentive to pathologists to teach and inform future doctors, policymakers, and patients about the perplexity of the pathology specialty. It shows that there are obvious gaps about the knowledge of the treatment process as a whole. It is hoped that doctors will take part in providing information to patients and family members, outweighing the deceptive information provided by the Internet. We hope that patients can be informed of the individual influences of each specialist treating them, including pathologists, and what they provide in creating the best treatment plan. Policymakers need to be aware of the significance pathologists may have in patient compliance and satisfaction to allow for proper funding. Pathologists should be open to clarifying questions patients have on social media allowing for better portrayal of the pathology specialty to younger generations. Better awareness of the importance of pathology to medicine and society is necessary to encourage young doctors to join the field of pathology. Doctors want to make an impact after so many years of studying and this is absolutely possible in the field of pathology. Pathologists need to maintain their historic role in patient care and remember that pathology is not about specimens, but about their current and future patients.
